# Vincamine Ameliorates Epithelial-Mesenchymal Transition in Bleomycin-Induced Pulmonary Fibrosis in Rats; Targeting TGF-β/MAPK/Snai1 Pathway

**DOI:** 10.3390/molecules28124665

**Published:** 2023-06-09

**Authors:** Rania Alaaeldin, Reham H. Mohyeldin, Amany Abdlrehim Bekhit, Wafaey Gomaa, Qing-Li Zhao, Moustafa Fathy

**Affiliations:** 1 Department of Biochemistry, Faculty of Pharmacy, Deraya University, Minia 61111, Egypt; rania.alaadin@deraya.edu.eg; 2 Department of Pharmacology and Toxicology, Faculty of Pharmacy, Deraya University, Minia 61111, Egypt; reham.hassan@deraya.edu.eg; 3 Department of Biochemistry, Faculty of Pharmacy, Minia University, Minia 61519, Egypt; amany_bkeet@mu.edu.eg; 4 Department of Pathology, Faculty of Medicine, Minia University, Minia 61519, Egypt; wafgom@mu.edu.eg; 5 Department of Radiology, Graduate School of Medicine and Pharmaceutical Sciences, University of Toyama, Toyama 930-0194, Japan; 6 Department of Regenerative Medicine, Graduate School of Medicine and Pharmaceutical Sciences, University of Toyama, Toyama 930-0194, Japan

**Keywords:** vincamine, pulmonary fibrosis, EMT, apoptosis, TGF-β1, MAPK, Snai1

## Abstract

Idiopathic pulmonary fibrosis is a progressive, irreversible lung disease that leads to respiratory failure and death. Vincamine is an indole alkaloid obtained from the leaves of *Vinca minor* and acts as a vasodilator. The present study aims to investigate the protective activity of vincamine against EMT in bleomycin (BLM)-induced pulmonary fibrosis via assessing the apoptotic and TGF-β1/p38 MAPK/ERK1/2 signaling pathways. In bronchoalveolar lavage fluid, protein content, total cell count, and LDH activity were evaluated. N-cadherin, fibronectin, collagen, SOD, GPX, and MDA levels were determined in lung tissue using ELISA. *Bax*, *p53*, *bcl2*, *TWIST*, *Snai1*, and *Slug* mRNA levels were examined using qRT-PCR. Western blotting was used to assess the expression of TGF-β1, p38 MAPK, ERK1/2, and cleaved caspase 3 proteins. H & E and Masson’s trichrome staining were used to analyze histopathology. In BLM-induced pulmonary fibrosis, vincamine reduced LDH activity, total protein content, and total and differential cell count. SOD and GPX were also increased following vincamine treatment, while MDA levels were decreased. Additionally, vincamine suppressed the expression of *p53*, *Bax*, *TWIST*, *Snail*, and *Slug* genes as well as the expression of factors such as TGF-β1, p/t p38 MAPK, p/t ERK1/2, and cleaved caspase 3 proteins, and, at the same time, vincamine increased *bcl2* gene expression. Moreover, vincamine restored fibronectin, N-Catherine, and collagen protein elevation due to BLM-induced lung fibrosis. In addition, the histopathological examination of lung tissues revealed that vincamine attenuated the fibrotic and inflammatory conditions. In conclusion, vincamine suppressed bleomycin-induced EMT by attenuating TGF-β1/p38 MAPK/ERK1/2/TWIST/Snai1/Slug/fibronectin/N-cadherin pathway. Moreover, it exerted anti-apoptotic activity in bleomycin-induced pulmonary fibrosis.

## 1. Introduction

Pulmonary fibrosis is an irreversible form of lung destruction with limited treatment options, eventually leading to organ dysfunction and disruption of gas exchange. Idiopathic pulmonary fibrosis (IPF) is a progressive form of lung fibrosis that leads to respiratory failure and death within five years, and the mortality rate is 50% [1]. IPF is characterized by severe production and deposition of the extracellular matrix (ECM) [2]. Moreover, IPF has been classified as a type II epithelial-mesenchymal transition (EMT) event in which the persistence of EMT-inducing signals leads to tissue remodeling and ECM accumulation [3].

EMT is a physiological process, whereas epithelial cells lose adhesive properties and gain some invasive mesenchymal features with further production of ECM [4,5]. EMT develops in response to carcinogenesis, injury, and fibrosis [6,7]. Notably, EMT can be detected by screening biomarkers that reflect the loss of epithelial characteristics and acquiring mesenchymal properties, such as the gain of N-cadherin, fibronectin [8].

Transforming growth factor beta 1 (TGF-β1) is an inflammatory mediator highly released in inflammatory and oxidative stress conditions [9,10]. It is up-regulated in the inflammation of lung tissues and is considered a hallmark of pulmonary fibrosis. Expressing TGF-β1 is also a critical regulator of EMT, which can activate EMT-dependent transcription factors such as TWIST, Snai1, and Slug [11]. Several in vitro studies indicated that TGF-β1 can induce EMT in human alveolar type II cells in a time and concentration-dependent manner [12,13]. Moreover, it showed a role in the trans-differentiation of quiescent fibroblasts to myofibroblasts [14]. Additionally, reactive oxygen species (ROS) can trigger the development of EMT via different signaling pathways [15].

Bleomycin, which is a chemotherapeutic agent commonly used in several cancers, including lymphoma, ovarian, testicular, and cervical cancer [16], was reported to cause pulmonary fibrosis via the production of ROS in young and old patients, even at low concentrations [17].

Vincamine is an indole alkaloid obtained from the leaves of *Vinca minor*. It showed a vaso-regulatory activity, exerted peripheral vasodilation and increased cerebral blood flow [18,19]. Many studies indicated its antioxidant, anti-inflammatory, and anti-apoptotic properties. Vincamine showed renal protection against cisplatin-induced nephrotoxicity [20]. In addition, it ameliorated the lipopolysaccharide-induced inflammation in human corneal epithelial cells [21] and attenuated the tamoxifen-induced oxidative stress [22]. Screening bioactive candidates for novel pharmacological effects is an attractive approach in the drug screening field [23,24,25,26,27]. Hence EMT and inflammation are essential processes during the pulmonary fibrosis, and targeting these pathways is crucial. Therefore, the present study aims to investigate the protective activity of vincamine against EMT in BLM-induced pulmonary fibrosis via assessing the apoptotic and TGF-β1/p38 MAPK/ERK1/2 signaling pathways.

## 2. Results

### 2.1. Total Cell Count, LDH Activity, and Total Protein Content

**Figure 1:**
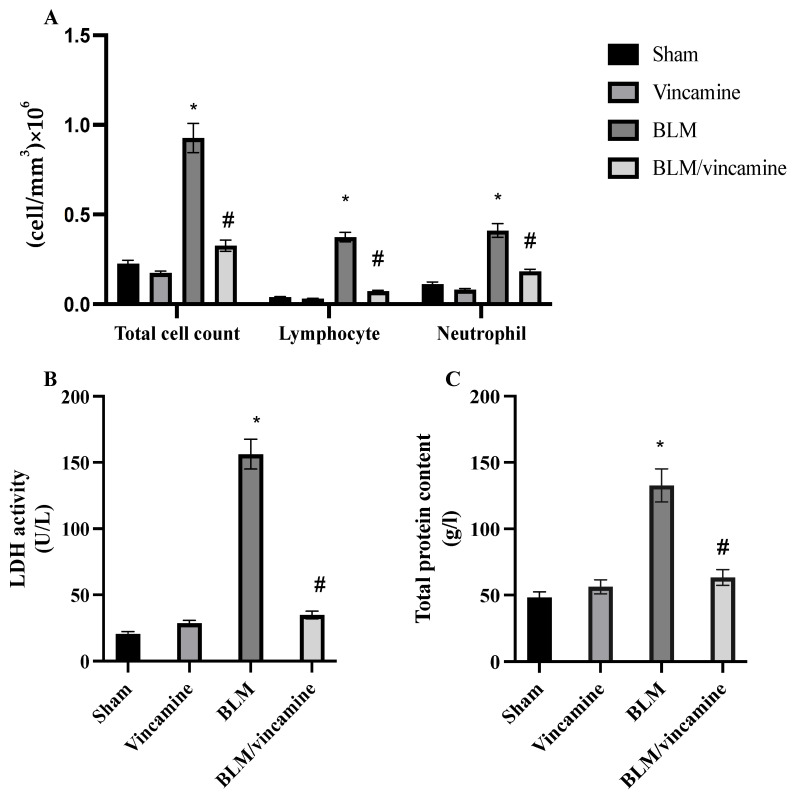
BALF analysis. Assessment of total cell count (**A**), LDH activity (**B**), and total protein content (**C**). Bars represent mean ± SD. Significant difference was analyzed by one-way ANOVA test followed by post hoc Dunnett test, where * *p* < 0.001, compared to sham group, and # *p* < 0.001, compared to BLM-induced group. BALF; Bronchoalveolar lavage fluid.

In order to evaluate lung deterioration with and without treatment with vincamine in BLM-induced groups, total and differential cell count, total protein content, and lactate dehydrogenase (LDH) activity were examined. As shown in Figure 1A, total cell count, lymphocytes, and neutrophils were significantly (*p* < 0.001) elevated in BLM-induced rats to 0.927 ± 0.081 cell/mm^3^, 0.374 ± 0.026 cell/mm^3^, and 0.411 ± 0.038 cell/mm^3^, respectively. While after vincamine treatment, total cell count, lymphocytes, and neutrophils significantly (*p* < 0.001) decreased to 0.327 ± 0.031 cell/mm^3^, 0.073 ± 0.005 cell/mm^3^, and 0.184 ± 0.011 cell/mm^3^, respectively.

Regarding LDH activity, BLM-induced rats showed increased (*p* < 0.001) levels of LDH to 156.3 ± 11.2 U/L, while vincamine treatment attenuated (*p* < 0.001) it to 34.83 ± 2.90 U/L, as shown in Figure 1B. Total protein content was also elevated (*p* < 0.001) in BLM-induced rats to 132.72 ± 12.50 g/L, whereas vincamine treatment decreased (*p* < 0.001) the total protein content to 63.3 ± 5.91 g/L, as shown in Figure 1C.

### 2.2. Measurement of Oxidative Stress

**Figure 2:**
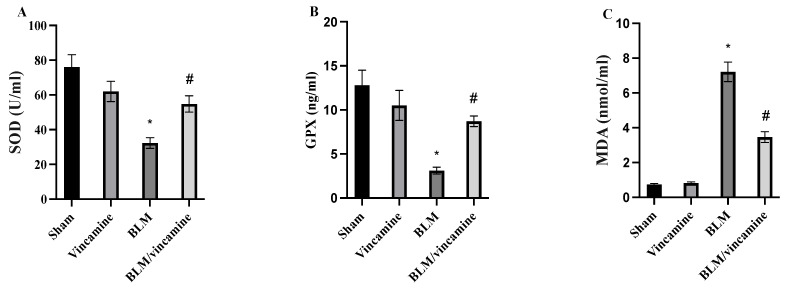
Lung tissue homogenates levels of SOD (**A**), GPX (**B**), MDA (**C**). Bars represent mean ± SD. Significant difference was analyzed by one-way ANOVA test followed by post hoc Dunnett test, where * *p* < 0.001, compared to sham group, and # *p* < 0.001, compared to the BLM-induced group.

Lung tissue levels of superoxide dismutase (SOD), glutathione peroxidase (GPX), and malondialdehyde (MDA) were examined to evaluate the oxidative stress status with or without treatment of vincamine in the BLM-induced groups. As shown in Figure 2A, SOD levels were decreased (*p* < 0.001) to 32.3 ± 3.1 U/mL in the BLM-induced group, compared to the sham group. While vincamine treatment elevated (*p* < 0.001) its levels to 54.8 ± 4.7 U/mL, compared to BLM-induced group. Regarding GPX levels, BLM-induced group showed a notable (*p* < 0.001) reduction in GPX levels to 3.1 ± 0.4 ng/mL, compared to the sham group, whereas vincamine treatment elevated (*p* < 0.001) its levels to 8.7 ± 0.6 ng/mL, compared to the BLM-induced group, as shown in Figure 2B. Also, MDA levels were significantly (*p* < 0.001) increased to 7.21 ± 0.56 nmol/mL in the BLM-induced group, compared to the sham group, while vincamine treatment significantly (*p* < 0.001) decreased its levels to 3.46 ± 0.31 nmol/mL, compared to the BLM-induced group, as shown in Figure 2C.

### 2.3. Evaluation of Fibronectin, N-Cadherin, and Collagen Levels

**Figure 3:**
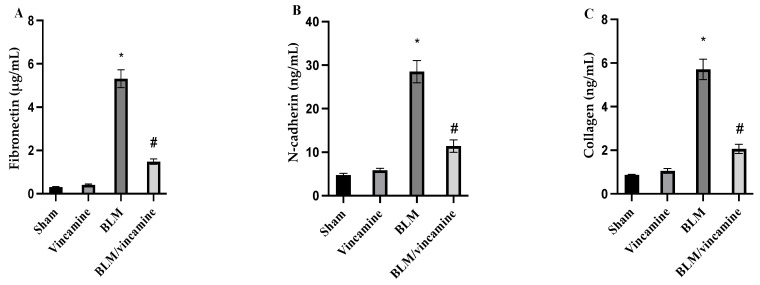
Effect of vincamine on fibronectin (**A**), N-cadherin (**B**), and collagen (**C**) levels in lung tissues. Bars represent mean ± SD. Significant difference was analyzed by one-way ANOVA test followed by post hoc Dunnett test, where * *p* < 0.001, compared to sham group, and # *p* < 0.001, compared to BLM-induced group.

Fibronectin, N-cadherin, and collagen levels were evaluated in the present study. As shown in Figure 3A, fibronectin levels showed notable (*p* < 0.001) increase in BLM-induced groups to 5.31 ± 0.41 μg/mL, compared to the sham group, while vincamine treatment significantly decreased (*p* < 0.001) fibronectin levels to 1.48 ± 12 μg/mL compared to the BLM-induced group. N-cadherin levels were significantly (*p* < 0.001) elevated to 28.5 ± 2.54 ng/mL in the BLM-induced group compared to the sham group, whereas vincamine treatment notably (*p* < 0.001) decreased N-cadherin levels to 11.37 ± 1.43 ng/mL compared to the BLM-induced group (Figure 3B). Additionally, collagen levels were elevated (*p* < 0.001) to 5.71 ± 0.47 ng/mL in BLM-induced groups compared to the sham group. Then, following vincamine treatment, collagen levels significantly decreased (*p* < 0.001) to 2.06 ± 0.21 ng/mL compared to the BLM-induced group, as shown in Figure 3C.

### 2.4. Gene Expression of p53, Bax, bcl2, TWIST1, Snai1, and Slug

**Figure 4:**
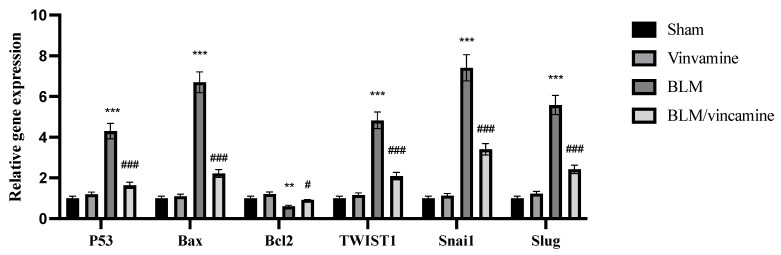
Expression of *p53*, *Bax*, *bcl2*, *TWIST1*, *Snai1*, and *Slug* genes in lung tissue homogenates. Quantitative RT-PCR was used to evaluate the gene expression of different groups. Expression was normalized to the corresponding *GAPDH* gene expression and expressed relative to the untreated sham group. Bars represent mean ± SD. Significant difference was analyzed by two-way ANOVA followed by post hoc Dunnett test, where ** *p* < 0.01 and *** *p* < 0.001 compared to the sham group, and # *p* < 0.05 and ### *p* < 0.001, compared to BLM group.

Expression of *p53*, *Bax*, *bcl2*, *TWIST1*, *Snai1*, and *Slug* genes was evaluated in the present study, as shown in Figure 4. mRNA levels of *P53*, *Bax*, *TWIST1*, *Snai1*, and *Slug* were significantly (*p* < 0.001) elevated in the BLM-induced group, compared to the sham group. While following vincamine treatment, their levels were notably (*p* < 0.001) suppressed compared to the BLM-induced group. On the other hand, expression of *bcl2* gene showed a notable (*p* < 0.01) decrease in the BLM-induced group compared to the sham group, whereas its mRNA levels were significantly increased (*p* < 0.05) following the treatment with vincamine compared to the BLM-induced group.

### 2.5. Expression of TGF-β1, p38 MAPK, ERK1/2, and Cleaved Caspase 3 Proteins

**Figure 5:**
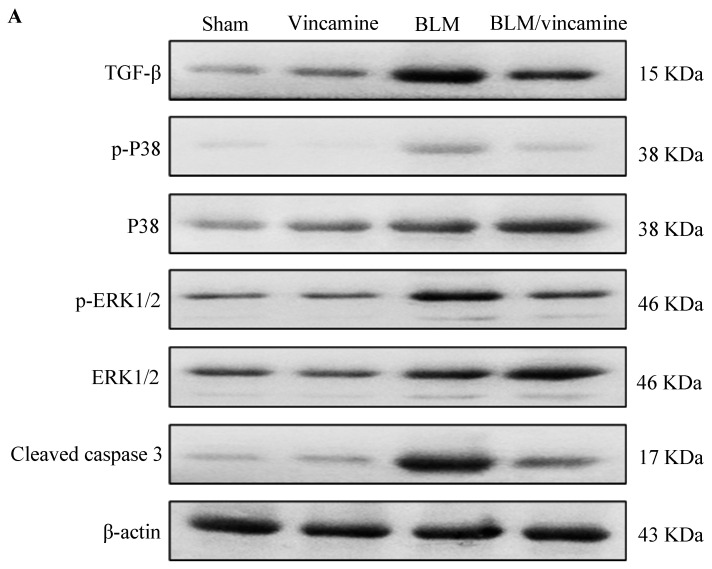

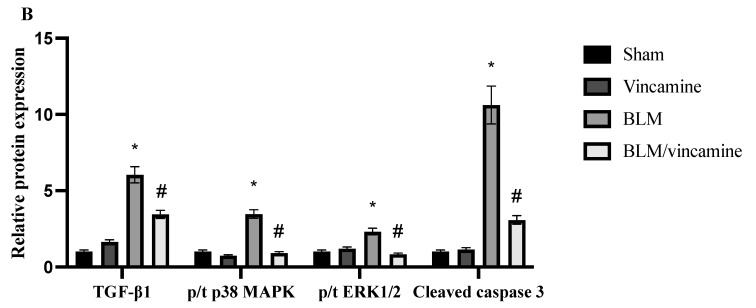
Effect of vincamine on the expression of TGF-β1, p38 MAPK, ERK1/2, and cleaved caspase 3 proteins. (**A**) Representative western blots of TGF-β1, p38 MAPK, ERK1/2, and cleaved caspase 3 proteins for different groups. (**B**) Expressions of TGF-β1, p/t p38 MAPK and p/t ERK 1/2 and cleaved caspase 3 proteins were expressed densitometrically, using bands in (**A**) after normalization to the corresponding internal control β-actin as fold change relative to that of sham control rats. Bars represent mean ± SD. Significant difference was analyzed by two-way ANOVA test followed by post hoc Dunnett test, where * *p* < 0.001, compared to sham group, and # *p* < 0.001, compared to BLM group.

As shown in Figure 5, compared to rats of the sham group, expression of TGF-β1, p/t p38 mitogen-activated protein kinase (p38 MAPK) and p/t extracellular-signal-regulated kinase 1/2 (ERK1/2) and cleaved caspase 3 proteins was notably (*p* < 0.001) elevated in the BLM-induced group. However, vincamine treatment substantially (*p* < 0.001) attenuated their expression compared to the BLM-induced group.

### 2.6. Histopathological Examination

#### 2.6.1. Protective Activity of Vincamine against Lung Inflammation Induced by BLM

**Figure 6:**
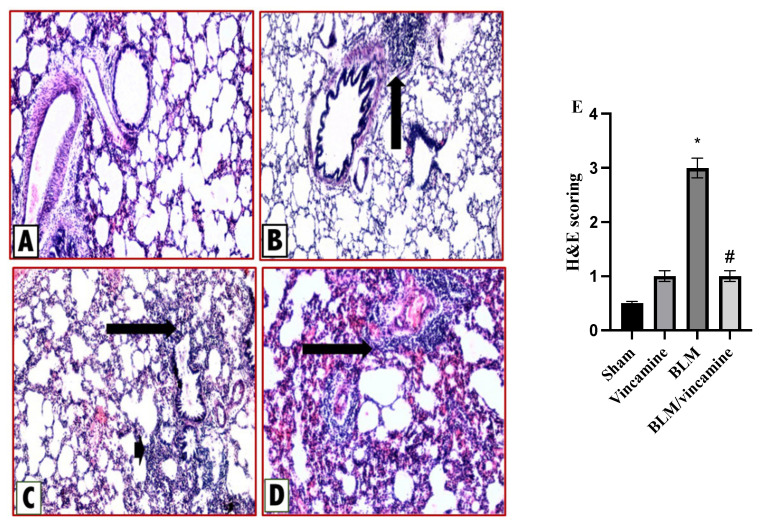
Activity of vincamine against lung inflammation induced by BLM. Representative photomicrographs of hematoxylin-eosin-stained lung tissues of different groups (magnification; 100×). (**A**) Sham group with histological features within normal limits, (**B**) Vincamine-treated group with a minimal chronic inflammatory infiltrate in alveolar septae (arrow), (**C**) BLM-induced group with marked thickening of the interalveolar septa (arrowhead) and peribronchiolar tissues (arrow) by chronic inflammatory cellular infiltrate., and (**D**) BLM/vincamine-treated group with moderate chronic inflammatory infiltrate was appeared in alveolar septae (arrow). (**E**) Lung inflammatory status histological score. Bars represent mean ± SD. Significant difference was analyzed by one-way ANOVA test followed by post hoc Dunnett test, where * *p* < 0.001, compared to sham group, and # *p* < 0.001, compared to BLM-induced group.

Lung tissue sections were stained with hematoxylin & eosin (H & E) to evaluate the inflammatory status with and without vincamine treatment as shown in Figure 6. Rats of the sham group (A) exerted normal lung histological appearance, while a minimal chronic inflammatory infiltrate in alveolar septae was found in vincamine-administered rats (B). In contrast, the BLM-induced group (C) exhibited obvious marked thickening of the interalveolar septa by chronic inflammatory cellular infiltrate. Finally, moderate alveolitis was represented in the BLM-treated group with vincamine (D) where a moderate chronic inflammatory infiltrate was found in alveolar septae. H & E scoring was determined (Figure 6E), whereas compared to the sham group, the BLM group showed a notable (*p* < 0.001) increase in inflammatory status. However, vincamine treatment significantly (*p* < 0.001) attenuated the inflammation compared to the BLM group.

#### 2.6.2. Protective Activity of Vincamine against Lung Fibrosis Induced by BLM

**Figure 7:**
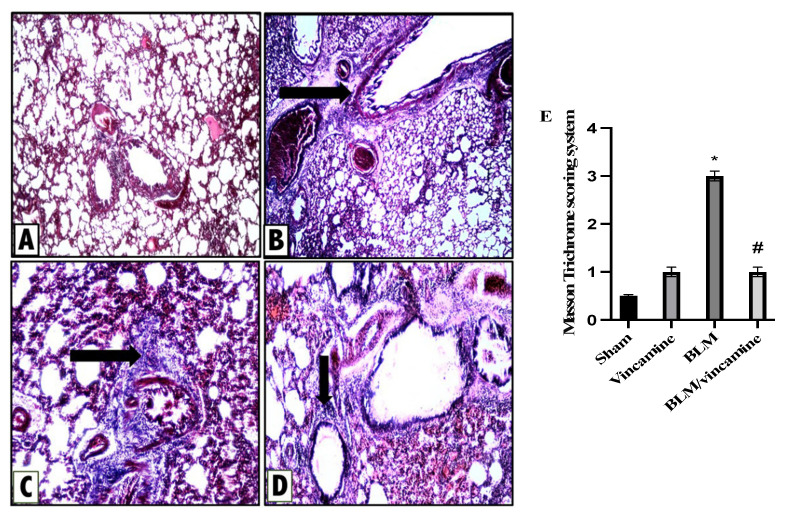
Activity of vincamine against lung fibrosis induced by BLM. Representative photomicrographs of Masson Trichrome stained lung tissues of different groups (magnification; 100×). (**A**) Sham group showing no fibrosis, (**B**) Vincamine-treated group with minimal fibrous thickening of peribronchiolar wall (arrow), (**C**) BLM-induced group with moderate thickening of peribronchiolar wall without obvious damage of lung architecture (arrow), and (**D**) BLM/vincamine-treated group with moderate fibrous thickening of peribronchiolar wall (arrow). (**E**) Lung fibrotic status histological score. Bars represent mean ± SD. Significant difference was analyzed by one-way ANOVA test followed by post hoc Dunnett test, where * *p* < 0.001, compared to sham group, and # *p* < 0.001, compared to BLM-induced group.

To examine the fibrotic status of lung tissue with and without vincamine treatment in BLM-induced groups, Masson Trichome staining was utilized as shown in Figure 7. Sham group rats (A) exerted normal lung histological appearance with no evidence of fibrosis, while a minimal fibrous thickening of peribronchiolar and alveolar walls was found in vincamine-administered rats (B). On the other hand, the BLM-induced group (C) exhibited marked fibrous thickening of peribronchiolar and alveolar wall without obvious lung architecture damage. Furthermore, moderate alveolitis was represented in the BLM-treated group with vincamine (D) where there was a moderate fibrous thickening of peribronchiolar and alveolar wall.

In addition, Masson Trichome scoring system was determined. Compared to the sham group, the BLM group showed significant (*p* < 0.001) elevation in the fibrotic status while vincamine treatment significantly (*p* < 0.001) reduced the fibrotic status compared to the BLM-induced group (Figure 7E).

## 3. Discussion

IPF is a heterogenous progressive pathological disease that is refractory to therapeutics. Replacing dead or damaged cells after an injury is critical for healing and survival. However, if the repairing mechanism is impaired, it can lead to the generation of fibrosis, characterized by the excess deposition of the ECM. Notably, fibrosis can be defined as an impaired healing process [28]. Lately, many attempts are ongoing, such as repurposing drugs [29,30,31,32] or looking for new bioactive synthetic [33,34,35,36] or natural [37,38,39,40,41] molecules that show promising therapeutic potential agents.

Vincamine, a herbal candidate, is a monoterpenoid indole alkaloid of clinical use, showing antioxidant, antibacterial, anti-inflammatory, and hypoglycemic activities [42]. Vincamine can potentially restore testicular steroidogenesis due to its antioxidant activity and protect against reproductive problems related to diabetes [43]. In addition, its biological properties seem to be related to the attenuation of the renal ischemia/reperfusion injury by suppressing apoptosis and inhibiting MAPK pathways [44]. In endolymphatic hydrops, it was also reported that vincamine exerted protective activities on spiral ganglion neurons and improved hearing through its antioxidant and anti-apoptotic activities [45]. Furthermore, it protected the corneal epithelial cells and attenuated the inflammation and oxidative stress induced by lipopolysaccharide [21]. Thus, the present study aimed to investigate the possible protective effect of vincamine against EMT in a BLM-induced pulmonary fibrosis model.

The present study examined the total protein content to investigate the destruction of alveolar epithelial cells after BLM induction. Total and differential cell count was also examined to estimate the degree of lung inflammation. Interestingly, vincamine attenuated the severity of alveolar and lung inflammation by reducing total protein content and decreasing the total and differential cell counts, neutrophil, and lymphocyte counts, respectively. Additionally, to assess interstitial lung damage, LDH activity was estimated [46]. Our findings revealed that BLM elevated the LDH activity, demonstrating increased interstitial lung injury, while vincamine treatment notably suppressed the LDH activity. These findings demonstrate that vincamine diminished interstitial lung damage in addition to the severity of alveolar and lung inflammation.

Oxidative stress and inflammation are the crucial hallmarks of lung fibrosis. Moreover, BLM was shown to induce oxidative stress and the generation of ROS in lung fibrosis [47]. In the present study of SOD and GPX activities, the cell antioxidant defense mechanisms were decreased by BLM while the lung MDA content was elevated. However, following the treatment with vincamine, SOD and GPX activities were notably increased and MDA content was reduced, indicating the antioxidant properties of vincamine.

TGF-β1 is a crucial growth factor that promotes the pathogenesis of lung injury and EMT during fibrosis in pulmonary diseases. The activation of the MAPK signaling pathway is an essential downstream of TGF-β1-mediated pathway that results in tissue inflammation and fibrosis in several diseases, including pulmonary fibrosis [48,49]. Indole alkaloids were shown to exert antifibrotic activity against TGF-β1 signaling pathway [50]. In the present study, we examined the potential activity of vincamine against TGF-β1 mediated p38 MAPK/ERK1/2 pathway before and after BLM induction. Our findings revealed that TGF-β1, p/t p38 MAPK, and p/t ERK1/2 proteins expressions were notably elevated by BLM, while vincamine effectively attenuated their expression in the fibrotic rats, suggesting the potential inhibitory activity of vincamine against TGF-β1/p38/ERK1/2 pathway and its ability to attenuate pulmonary EMT which is induced by BLM. Our findings are in accordance with Lu et al., who reported that TGF-β1 induced the activation of the ERK signaling pathway, which is required for EMT development [51]. Another study by Witte indicated that inhibition of TGF-β1-dependent EMT occurred by inhibiting p38/MEK/ERK signaling pathway [52].

For further confirmation that vincamine can modulate EMT in BLM-induced pulmonary fibrosis in the present study, the expression of *TWIST*, *Snai1*, and *Slug* genes was measured in addition to the levels of fibronectin, N-cadherin, and collagen proteins.

*TWIST*, *Snai1*, and *Slug* are transcription factors that were found to endure in tumor cells with stem cell characteristics and play a crucial role in EMT events [53]. Fibronectin and N-cadherin primarily indicate mesenchymal characteristics of the cells [54,55], and collagen is a major sign for the development of pulmonary fibrosis [56]. Our findings revealed that the mRNA levels of *TWIST*, *Snai1*, and *Slug* were up regulated by BLM, while vincamine treatment decreased their gene expression. Additionally, fibronectin, N-cadherin, and collagen were found to be elevated in rats treated with BLM. However, vincamine attenuated these levels following treatment, suggesting the promising action of vincamine in attenuating EMT and its potentiality in modulating pulmonary fibrosis.

Furthermore, to evaluate the apoptotic status of cells during pulmonary fibrosis in the present study, we detected the expression of *p53*, *Bax*, and *bcl2* genes and the protein levels of cleaved caspase 3. After BLM administration, mRNA levels of *p53* and *Bax* as well as cleaved caspase protein levels, all apoptotic factors, were substantially elevated, while *bcl2* mRNA level, an anti-apoptotic factor, was notably reduced. In this regard, vincamine treatment modulated these levels, indicating its significant anti-apoptotic activity.

For further confirmation of all previous analyses, histological examination supported our hypothesis, whereas H & E and Masson trichome staining were used to evaluate the inflammation and fibrosis status, respectively. In the present study, the BLM-induced group exerted increased inflammatory and fibrotic states of lung tissues, whereas vincamine treatment relieved these inflammatory and fibrotic states.

Further in vivo studies are required to confirm the mechanistic role of vincamine during IPF before starting clinical trials.

## 4. Materials and Methods

### 4.1. Drugs and Chemicals

Vincamine and BLM vials were supplied by Sigma Aldrich (#1617-90-9, Sigma-Aldrich, Inc., St. Louis, MO, USA) and (#B1141000, Sigma-Aldrich, Inc., St. Louis, MO, USA), respectively. Vincamine was administrated as a suspension in 0.5% carboxymethyl cellulose (CMC).

### 4.2. Experimental Animals

Study protocols and animal care were followed according to the guidelines established by The Experimental Animal Center and Research Ethics Committee (Approval Number: ES06/2021), Minia University, Minia, Egypt. Male Wistar rats aged 7–8 weeks and weighing (180–200 g) were obtained from Animal Research Center, Faculty of Medicine, Minia University. Separate cages were used for animal housing, supplied with fresh drinking water and commercial pellets for feeding, and kept in 12 h of light/dark cycles.

### 4.3. Pulmonary Fibrosis Induction

To anesthetize animals, pentobarbital sodium (50 mg/kg) was administrated intraperitoneally (i.p.). To expose the trachea of each rat, a vertical incision in the neck midline was made, whereas a single intratracheal injection of BLM (5 mg/kg in 0.9% NaCl) was administrated slowly to induce pulmonary fibrosis [57]. The sham group received an equal amount of sterile 0.9 NaCl in a similar manner. To ensure homogenous dispersion of BLM dose within the lung, rats were laid, gently massaged, and rotated several times [58]. Then, ketoprofen (3 mg/kg IM) was administrated as an analgesic to rats after the procedure [59]. The surgical incision was sutured, and iodopovidone was applied topically to sterilize the wound area.

### 4.4. Experimental Groups

Rats were randomly divided into four groups, each of 8 rats:

Group I (Sham group): An intratracheal injection of 0.9 NaCl was administrated on day 7 to animals. CMC was also orally administered to rats daily for 9 days.
Group II (Vincamine group): Animals orally administrated (40 mg/kg) vincamine [20] daily from day 1 till the end of the experiment.
Group III (BLM group): A single intratracheal injection of BLM (5 mg/kg in 0.9% NaCl) [57] was administrated to animals on the seventh day.
Group IV (BLM-treated group with vincamine): BLM-induced animals were pretreated with vincamine (40 mg/kg) daily 7 days prior to BLM injection and till the end of the experiment [60].

The experiment was extended for 9 days. The injection of BLM was performed on the seventh day and animal sacrifice occurred on day 9. According to a previously published study, the dosage and duration of vincamine were performed [20]. With urethane (25%, 1.6 g/kg, i.p) [61], rats were anesthetized at the end of the experiment. From the thoracic cavity, bronchoalveolar lavage fluid (BALF) was collected. To lavage the right lung lobe, 3 mL of 0.9% sterile saline (1 mL/time) was used thrice slowly. To obtain BALF, lungs were compressed to recover about 50–70% of the initial injected volume. The fluid was collected using centrifugation at 3500 rpm at 4 °C for 10 min, and the supernatant was stored for further analyses. In contrast, the sedimented cell pellets were analyzed to determine the differential and total cell counts. The right lung lobes were quickly collected and stored at −80 °C for further analysis. The left lungs were collected and immersed in formalin solution (10%) for histopathological and immunostaining examination.

### 4.5. Cell Count, LDH Activity, and Total Protein Content Evaluation

Quantification of neutrophils and lymphocytes was obtained by analyzing total and differential cell counts. BALF was centrifugated, and sedimented cells were collected and suspended in 500 mL sterile saline. Then, cells were stained with Wright-Giemsa for 8 min. 200 cells/slide were counted at 40× magnification, and the results were obtained as the number of cells/mm^3^.

In BALF, the activity of LDH enzyme and total protein concentration were also evaluated, according to manufacturer’s instructions, utilizing rat LDH ELISA kit (#MBS269777, MyBioSource, San Diego, CA, USA) and a protein determination kit (Spinreact Co., Girona, Spain), respectively.

### 4.6. ELISA Analysis

Levels of SOD, GPX, and MDA were evaluated in lung tissue homogenates utilizing (#MBS036924, MyBioSource, CA, USA), (#MBS744364, MyBioSource, CA, USA), and (#MBS268427, MyBioSource, CA, USA), respectively, according to the manufacturer’s instructions.

In lung tissue homogenates, levels of fibronectin, N-cadherin, and collagen were also assessed, according to the manufacturer’s instructions, utilizing rat fibronectin ELISA kit (#MBS761397, MyBioSource, CA, USA), N-cadherin ELISA kit (#KOA0665, Rockland immunochemical Inc., Royersford, PA, USA), and collagen type I ELISA kit (#MBS262647, MyBioSource, CA, USA), respectively.

### 4.7. Quantitative Real-Time Polymerase Chain Reaction

According to the Qiagen RNA extraction kit, lung tissue homogenates were utilized to extract total RNA (Hilden, Germany). mRNA levels of *TWIST1*, *Snai1*, *Slug*, *p53*, *Bax*, and *bcl2* genes were evaluated by real-time qPCR. mRNA quantification was performed by utilizing the Rotor-Gene 6000 Series Software 1.7. *Glyceraldehyde 3-phosphate dehydrogenase (GAPDH)* was utilized as internal control [62]. Primers’ sequences, mentioned in Table 1, were obtained from the National Centre for Biotechnology Information (NCBI). Using the Qiagen one-step RT-PCR (Qiagen), RT-PCR reactions were performed containing total RNA (100 ng), forward and reverse primers (0.6 μM), 1× buffer, dNTP (each of 400 μM), and an enzyme mix (2 μL). The conditions were 35 cycles of denaturation step 25 s at 95 °C, primers annealing 30 s at 58 °C, and a polymerization step for 20 s at 72 °C.

For each sample, real-time PCR reactions were performed in triplicate. The average cycle threshold was calculated for each sample. With the Rotor-Gene 6000 Series Software 1.7, in order to exclude the production of non-specific compounds, a melting curve analysis was obtained at 1 °C intervals between 60–95 °C using the SYBR Green fluorescent dye. After normalization to *GAPDH* expression and relative to the untreated sham group, the expression of genes in the treated groups was calculated.

### 4.8. Western Blotting Analysis

Expression of phosphorylated p38 MAPK, total p38 MAPK, phosphorylated ERK1/2, total ERK1/2, transforming growth factor beta (TGF-β1), and cleaved caspase 3 proteins was detected utilizing sodium dodecyl sulphate–polyacrylamide gel electrophoresis (SDS-PAGE) analysis.

Protein was extracted using RIPA lysis buffer from lung tissues containing Tris–Cl (50 mM), pH 7.5, Nonidet P-40 (1%), NaCl (150 mM), PMSF (1 mM), sodium deoxycholate (0.5%), and SDS (0.1%), boosted with the complete protease inhibitor cocktail (Roche, Mannheim, Germany). Sample protein concentrations were determined using the Bradford method. Protein samples (30 μg), in Blocking Solution, were incubated at room temperature for 1 h after the transfer to a Hybond™ nylon membrane (GE Healthcare). At 4 °C overnight, membranes were incubated with antibodies of p-p38 MAPK (#sc-166182, Santa Cruz Biotechnology, Inc., Dallas, TX, USA), p38 MAPK (#sc-81621, Santa Cruz Biotechnology, Inc., TX, USA), pERK1/2 (#sc-7383, Santa Cruz Biotechnology, Inc., TX, USA), ERK1/2 (#sc-514302, Santa Cruz Biotechnology, Inc., TX, USA), TGF-β1 (#sc-130348, Santa Cruz Biotechnology, Inc., TX, USA), cleaved caspase 3 (#YPA2268, BioSpes, Chongqing, China), and b-actin (#E-AB-20031, Elabscience, UK) diluted (1:1000) with PBS. Then, after washing, membranes were incubated for 1 h with the HRP-conjugated secondary antibody (New England Biolabs, Hertfordshire, UK) diluted (1:1000) in PBS at room temperature [63]. According to the manufacturer’s instructions of the enhanced chemiluminescence kit (GE Healthcare, Little Chalfont, UK), bands were detected, using a luminescent image analyzer (LAS-4000, Fujifilm Co., Tokyo, Japan). Electrophoresis and electroblotting, using a discontinuous buffer system, were carried out in a Bio-Rad Trans-Blot SD Cell apparatus (Bio-Rad, Hercules, CA, USA). Relative to the untreated sham group, Image Processing and Analysis Java (ImageJ, 1.8.0_172) program was used to perform the densitometric analysis after the normalization to β-actin levels.

### 4.9. Pathological Examination

Lung tissues paraffin sections (5 µm thickness) were divided into two groups. The first group was used for H & E staining for the inflammation assessment as previously mentioned [64], Table 2.

The second group was used for Masson trichrome staining for fibrosis assessment using the scoring system described in Table 3 [65].

### 4.10. Statistical Analysis

To analyze the differences of multiple comparisons, analysis of variance (ANOVA) followed by post hoc Dunnett test were used using GraphPad Prism 9 statistical software (GraphPad, La Jolla, CA, USA). Data were represented as mean ± standard deviation (SD). When *p* values < 0.05, differences were considered significant.

## 5. Conclusions

In conclusion, vincamine showed promising a protective effect against EMT in BLM-induced pulmonary fibrosis through its antioxidant and anti-apoptotic activities. It activated the antioxidant status by elevating SOD and GPX activities. Furthermore, it exerted its anti-apoptotic activity via inhibiting p53/Bax/cleaved caspase 3 pathway. Moreover, vincamine attenuated EMT by suppressing TGF-β1/p38 MAPK/ERK1/2/TWIST/Snai1/Slug/fibronectin/N-cadherin pathway.

## Figures and Tables

**Table 1:** Primers’ sequences.

Primer	Sequence of the Primer
*TWIST1*	Forward: 5′-CTCAGCTACGCCTTCTCCGT-3′ Reverse: 5′-TGACATCTAGGTCTCCGGCCT-3′
*Snai1*	Forward: 5′-ACCTCCAGACCCACTCGGAT-3′ Reverse: 5′-GAGGTAGCAGGGTCAGCGAG-3′
*Slug*	Forward: 5′-ATTCCTGGTGCGTGTCCCAT-3′ Reverse: 5′-GCAACGTGTGGGTCCGAATG-3´
*P53*	Forward: 5′-AGCGACTACAGTTAGGGGGT-3′ Reverse: 5′-ACAGTTATCCAGTCTTCAGGGG-3′
*Bax*	Forward: 5′-CGTCTGCGGGGAGTCAC-3′ Reverse: 5′-AGCCATCCTCTCTGCTCGAT-3′
*Bcl2*	Forward: 5′-TTCTCTCTTTCGGGCCGTGG-3′ Reverse: 5′-CCACTCGTAGCCCCTCTGTG-3′
*GAPDH*	Forward: 5´-AACCTGCCAAGTATGATGACATCA-3′ Reverse: 5′-TTCCACTGATATCCCAGCTGCT-3′

**Table 2:** The lung tissues alveolitis grading system.

Severity Score	Histology
(0) None	No
(1) Mild	<20% of the tissue
(2) Moderate	20–50% of the tissue
(3) Severe	>50% of the tissue

**Table 3:** The lung tissues fibrosis assessment grading system.

Fibrosis Score	Histological Findings
0 1	Normal Lung
	Minimal fibrous thickening of bronchial or alveolar walls
2 3	Moderate walls thickening without clear lung architecture damage
4 5	Increased lung structures fibrosis definite damage with formation of small fibrous masses or fibrous bands
6 7	Severe structure distortion and great areas of fibrosis (honeycomb lung)
8	Total field fibrous obliteration

## Data Availability

All data are fully available and included in the manuscript.
